# Learning Collaboratives as a Community-Organizing Strategy to Improve Heart Health

**DOI:** 10.5888/pcd23.250398

**Published:** 2026-08-20

**Authors:** Callie Bradley, Suzanne Grossman, Kendall Farr, Michele King, Victoria Sanders, Hamilton Peoples, Patrick Wiggins

**Affiliations:** 1 Institute for Innovation in Health and Human Services, James Madison University, Harrisonburg, Virginia; 2 Office of Family Health Services, Division of Prevention and Health Promotion, Virginia Department of Health, Richmond, Virginia

## Abstract

Cardiovascular disease and other chronic diseases contribute substantially to illness, death, and health inequities in the US. Despite medical advances and interventions to improve the health outcomes among people with chronic diseases, additional strategies are needed. Communities are a powerful driver of health promotion and disease prevention. When engaged meaningfully, communities can enact and sustain health-related changes starting at the individual level and expand these changes to system and policy levels. Grounded in the social action model and the principles of shared governance, learning collaboratives can promote community engagement, mutual accountability, and local capacity building. By aligning public health efforts with community priorities, learning collaboratives offer a way to build community trust, deepen community partnerships, and support sustainable health initiatives. They can position communities not merely as sites of intervention but as meaningful change agents in addressing health. This article explores the use of learning collaboratives, and in particular, the Healthy Hearts Learning Collaboratives in Virginia, as practical tools for advancing community-driven strategies to reduce the incidence of chronic disease and poor health outcomes. It provides guidance for entities looking to implement learning collaboratives focused on improving health and ensuring that people with lived experience have an opportunity to contribute to improving conditions to promote health in their communities.

**Summary**

**What is already known on this topic?**

Cardiovascular disease and other chronic diseases contribute substantially to illness, death, and health inequities in the US. Despite medical advances and interventions to improve health outcomes among people with chronic disease, additional strategies and tools are needed.

**What is added by this report?**

By aligning public health efforts with community priorities, learning collaboratives, such as the Healthy Hearts Learning Collaboratives in Virginia, offer a way to build community trust, deepen community partnerships, and support sustainable health initiatives. These strategies can improve health outcomes associated with cardiovascular disease and other chronic diseases.

**What are the implications for public health practice?**

Learning collaboratives can be used as a tool to promote healthy lifestyles in local communities.

## Introduction

Heart disease is the leading cause of death in the US (1). Accordingly, improving cardiovascular health and reducing the number of deaths from heart disease and stroke is a goal for Healthy People 2030 (2). Due to the complexity of heart disease, its multiple contributing factors, and the long-term, often chronic nature of heart disease, preventing, managing, and treating it is challenging. Many risk factors associated with heart disease are shaped by social, environmental, and structural conditions that extend beyond the reach of medical care (3). This broader context highlights that comprehensive, multidisciplinary approaches, including community action, are required to improve cardiovascular health outcomes.

The socioecological model shows how health outcomes, such as those associated with cardiovascular disease, are influenced by intrapersonal, interpersonal, community, and societal factors (3). At the intrapersonal level, individual lifestyle choices, such as poor diet and physical inactivity, can increase risk for cardiovascular disease. Yet lifestyle choices are often influenced by interpersonal factors. For example, strong social connections with healthy role models may influence a person’s likelihood of exercising or eating well and may provide the necessary resources, support, and encouragement for making healthier choices. Communities are influenced by larger societal factors such as culture, history, built environments, and policies. Health strategies that focus only on the intrapersonal level fail to capture the importance of influences of interpersonal relationships and one’s community.

A thriving community is one where people work together collectively to address what matters most to them, whether reducing violence, revitalizing neighborhoods, or promoting health (4). When a community is strong, it can play a lead role in addressing public health problems. Community strength and health are shaped by social determinants of health; barriers related to education, employment, and housing contribute to disparities and poor health outcomes (5). Interventions at the community level are effective at addressing social determinants of health and chronic disease management because they allow people to come together to learn techniques to support healthy lifestyles as well as advocate to local, state, and federal lawmakers for policy and community change (eg, neighborhood parks) (6).

Communities can be leveraged as a tool to address major public health concerns if key community members support this mission. This article outlines how learning collaboratives, developed by using the social action model and principles of shared governance as a framework, can empower communities to take meaningful action toward preventing cardiovascular disease and other chronic diseases. We offer details and examples that draw from our experiences developing and implementing the Healthy Hearts Learning Collaboratives, which comprise community-led groups that bring together residents, health care organizations, and community partners to address local cardiovascular health needs in urban and rural communities in Virginia. Using a community-organizing approach, these collaboratives identify priorities, develop action plans, and implement strategies to improve heart health and reduce disparities in their communities. 

## Development and Integration of Learning Collaboratives

### Overview

Learning collaboratives can be defined as a group of public health entities, health systems, health care providers, and community leaders who come together with a common goal of implementing evidence-based initiatives to improve the health of a community (7). Learning collaboratives can be used to advance health equity initiatives and reduce systemic barriers through shared goals, community-driven approaches to foster trust and relevance, and amplification of community voices (6,8,9). Unlike other community-based strategies, which are often set in specific sites, such as schools and workplaces (10), and reflect the experiences and perspectives of that site only, learning collaboratives intentionally convene members across a wider spectrum. This collaborative approach promotes shared decision-making and leverages different experiences and perspectives to address complex issues (11).

### Guiding framework

Alinksy’s social action model for community organizing promotes the use of education, advocacy, and collective effort to address change (12). Following this model ensures that community voices and shared governance are at the forefront of a learning collaborative’s guiding structure (13). Community organizing builds local power by creating cohesive organizations that represent communities and enable them to advocate effectively with decision makers (14). A component of community organizing is the development of Freire’s critical consciousness, defined as the ability to recognize social, political, and economic contradictions and to act against the oppressive elements of reality (15). Critical consciousness aims to raise awareness of inequities, which, if unrecognized, can perpetuate health disparities and increase disease risk and prevalence (16).

Community organizing is more likely to succeed when critical consciousness is achieved and actions are driven by community priorities and desire for change, rather than externally imposed agendas (7,17). Community organizing combined with embedded principles of critical consciousness aligns with the socioecological model by recognizing the multiple levels that influence health and integrating strategies from theories of social networks, social support, and social systems (18). This combined approach allows learning collaboratives to create a participatory, shared governance environment where learners are active and empowered co-creators of their health.

Following Freire’s pedagogy that emphasizes critical consciousness, learning collaborative members can engage in real-world discussions and critical reflection of the health status in their communities while developing solutions through collective action (15). Cultivating critical consciousness for advocacy requires intentional support for capacity building and does not simply call for participation. Learning collaboratives that include community health workers, structured facilitation, and ongoing technical assistance can equip communities with the knowledge, leadership skills, and shared governance structures necessary to navigate and address structural barriers collectively (19).

**Figure 1:**
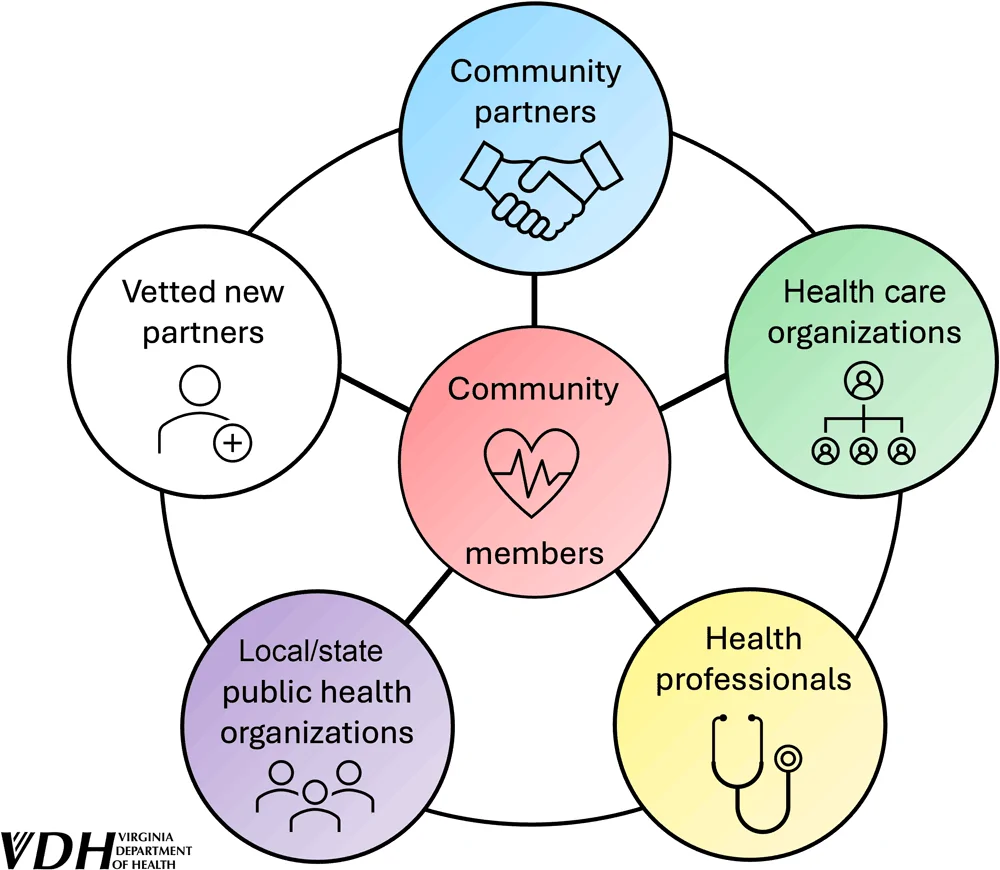
The Healthy Hearts Learning Collaboratives, Virginia, a model for a learning collaborative.

Merging critical consciousness and community organizing facilitates a process of mutual grassroots action where community members, health systems, and public health organizations can come together, build trust, and empower people to take ownership of their health. By aligning with the principles of shared governance, which emphasize participatory decision-making (20), learning collaboratives provide both a supportive environment that can empower community members to take part in public health decision-making and a platform to amplify their voices to promote positive change from the ground up. Community members are at the heart of the learning collaborative framework (Figure 1).

## Implications for Practice

The following steps, based on the theoretical framework of Alinsky’s community organizing and Freire’s pedagogy, could be used to create learning collaboratives that prioritize community voice and shared governance for health initiatives and advocacy.

### Preparation

To initiate the development of a successful learning collaborative, an organizer needs to emerge from the community to convene residents and begin the process of collectively determining the learning collaborative’s mission, vision, and goals. To start this process, initial learning collaborative organizers (eg, highly engaged and motivated community members) should be educated in humble leadership practices such as engaging in shared decision-making and active listening, using empathetic reflective language, and demonstrating genuine interest in learning what the community has to say.

The Healthy Hearts Learning Collaboratives, initially funded in 2023 through the Centers for Disease Control and Prevention via the Virginia Department of Health, were initiated after interested community members were recruited by a coordinator funded through the Virginia Department of Health to form a learning collaborative in their communities. Each of the 8 Healthy Hearts Learning Collaboratives functions independently; each has its own mission, vision, goals, and projects. Membership recruitment occurred through discussing the concept of a learning collaborative with community members and leveraging existing learning collaborative member networks, trusted referrals, and connections with local organizations. Learning collaborative organizers also embedded themselves in community spaces (eg, attending local classes and events) to build visibility, trust, and transparency.

When establishing learning collaborative membership, focusing on intentional relationship building, authenticity, and social capital while emphasizing a shared purpose is critical. Learning collaboratives should be composed ideally of approximately 12 to 15 members, although the Healthy Hearts Learning Collaboratives often began with 5 to 8 and expanded over time. While organizational representation may vary, partners should be intentionally selected to advance the learning collaborative’s work while maintaining a structure that uplifts community voices and power. All prospective Healthy Hearts members were vetted; they completed a structured interview process, which included assessing social capital, fit, and alignment with learning collaborative values. Early members identified roles based on individual strengths, and leaders were selected to advance specific activities. This approach ensured that projects reflected lived experience, leveraged community insight, and addressed local needs.

### Foundational development, implementation and evaluation, and sustainability

**Figure 2:**
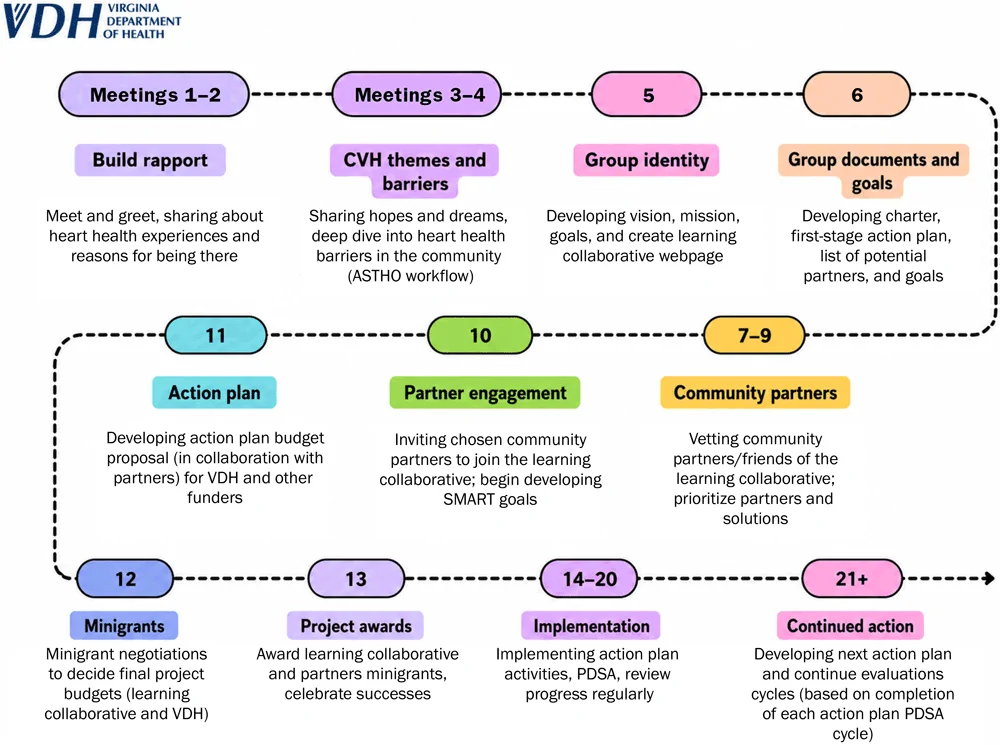
Timeline for the Healthy Hearts Learning Collaboratives, Virginia. Abbreviations: ASTHO, Association of State and Territorial Health Officials; CVH, cardiovascular health; PDSA, plan-do-study-act; SMART, specific, measurable, actionable, realistic, and time-based; VDH, Virginia Department of Health.

The next step was to begin developing each learning collaborative’s identity, infrastructure, and action plan, while mobilizing community assets to implement activities. Creating a timeline and plan is important, because not all desired activities are immediately achievable. We developed a timeline of best practices for meeting development and delivery objectives, which was then distributed to learning collaborative coordinators (Figure 2). This timeline highlights the importance of flexibility, respect for the community, and commitment to aligning mutual goals to build trust and ensure that the community remains at the heart. These steps can be iterative, with learning collaboratives and communities revisiting any step to ensure sustainability.

#### Foundational development

Because laying a strong foundation is critical in the early stages of developing a learning collaborative, early meetings should focus on the following key elements.

**Building rapport.** Easing communication involves forming relationships characterized by mutual understanding and empathy. Selected community members can be convened for casual meet-and-greets during initial meetings. Holding meetings at convenient times and neutral locations (eg, public library, social services building) can create a welcoming environment.

**Health themes and barriers.** The next step is to identify common themes and experiences of the root causes and barriers to good health, acknowledge learning collaborative members’ lived experiences of these causes and barriers, and appreciate how discussing these experiences can create shared knowledge and tools for advocacy. Group discussions can be held to work through this process.

**Group identity.** During the first several meetings, learning collaborative members should be guided through a process to develop a shared vision, mission, and set of goals based on the themes, barriers, and collective hopes discussed, which should then be disseminated on a webpage.

**Group documents and goals.** Once a group identity is established, developing a charter and first-stage action plan is important to solidify and share learning collaborative goals and objectives with the broader community. This process can be conducted through direct outreach via social networks, neighborhood newsletters, community surveys, and other avenues of communication. For example, for Healthy Hearts, the state health department, as a core partner, created a webpage for each learning collaborative, outlining the mission, vision, goals, and progress of each.

**Community partners and partner engagement.** After establishing grassroots momentum, the learning collaborative should invite prospective community partners to their meetings as part of a vetting process to determine alignment with the learning collaborative’s mission and the partners’ potential contributions. These partners can include local elected officials and members of community organizations who can help advocate for and meet the health-related needs identified (eg, concerns about transportation, access to healthy foods). To ensure authentic community power, at least half of learning collaborative members should be community residents who have lived experience of a particular health problem and who actively participate in shared governance and decision-making. The remaining members may include community partners who support the learning collaborative’s mission and goals but do not participate in the governance structure.

**Action plan.** An action plan describes the goals of the learning collaborative in terms of actions to be taken, such as assigning partners to lead activities, determining a timeline, creating a budget, and developing proposals for potential funders. At Healthy Hearts, during several conversations, one learning collaborative developed specific, measurable, actionable, realistic, and time-based (SMART) goals to address food insecurity, exercise and nutrition education, and blood pressure self-monitoring, all focused on building on community strengths and addressing locally identified needs.

**Securing funding.** Learning collaboratives can secure financial support by engaging local partners who are willing to provide financial assistance on certain initiatives, particularly when the effort is community-led and aligns with the partners’ mission. Meeting with potential funders allows learning collaborative members to share their vision, demonstrate how funders can contribute, and make a direct ask for funding. Identifying prospective local, state, and national funders whose values align with the learning collaborative’s mission is essential for sustaining activities. Leveraging local resources (eg, universities, public libraries), learning collaborative networks can identify grant opportunities beyond traditional academic or government entities. For example, Healthy Heart learning collaboratives partnered with local organizations to serve as fiduciary agents, implement programs, and provide a venue for community services.

#### Implementation and Evaluation

Once a strong foundation has been created, the learning collaborative moves into the implementation phase. The first activities implemented by the Healthy Hearts Learning Collaboratives were organizing walking clubs, working with the city and local health department to implement evidence-based lifestyle change programs, and holding events at community hubs (eg, churches, senior centers) where prospective learning collaborative members could be recruited. Later-stage activities included incorporating a community health worker to aid in screening community members for cardiovascular health risks and making referrals to health care resources. In addition, learning collaboratives collected and reviewed community feedback, participation metrics, and outreach data to identify emerging needs, assess progress, and guide data-driven quality improvement efforts. One model for evaluating the effectiveness and impact of a learning collaborative is the Plan-Do-Study-Act cycle. These cycles are iterative quality improvement processes that test and refine change strategies in real time based on what is learned. For example, one Healthy Hearts Learning Collaborative recognized it was struggling to meaningfully connect with the community and identify needs. The learning collaborative planned and implemented surveys and door-to-door outreach (Plan), engaged in one-on-one conversations (Do), assessed the feedback gathered (Study), and refined its goals and strategies to better align with expressed community priorities (Act).

#### Sustainability

Building sustainability into community-centered public health programs is essential to ensure long-term impact and maintain community trust. Sustainability can take the form of building relationships with local businesses (such as grocery stores and gyms), securing stable funding and in-kind donations, and maintaining engagement among partners. One Healthy Hearts Learning Collaborative secured a partnership with a local community center to dedicate a permanent space for learning collaborative activities and meetings, which allowed the learning collaborative to continue operations without concern over venue or rental fees. Shared governance in developing and implementing action plans also helps to ensure that learning collaboratives can navigate through challenges such as changes in leadership and membership. Together, these elements lay the foundation for resilient, community-driven health improvements that can be maintained and scaled over time.

## Recommendations

Learning collaboratives can be used as an evidence-based health promotion tool that can influence all levels of society: individual, interpersonal, institutional, community, and policy. With intentional design, development, and implementation, learning collaboratives can serve as an instrument for change in addressing health, although further longitudinal research is needed to quantify their influence. The following recommendations are given as a starting point and guide for community implementation of a learning collaborative.

### Individual

At the individual level, learning collaboratives are a tool for empowerment. People often join a learning collaborative to change patterns in their own health and wellness. Knowing this, learning collaboratives should leverage a person’s skills, talents, and networks to foster that person’s empowerment while expanding the impact of the learning collaborative. Cultivating a person’s sense of critical consciousness, empathy, and active listening provides a strong shared governance foundation for building healthier lifestyles.

### Interpersonal

Interpersonally, learning collaboratives should focus on building social capital by encouraging diversity of thought, ideas, and talents. Membership should be inclusive and intentional, allowing anyone interested in improving health to join after being vetted by the learning collaborative. By fostering mutual respect and trust among members, learning collaboratives can create strong interpersonal networks that support collective health goals. Learning collaborative leaders and organizers should actively engage with members of the broader community to understand their needs and perspectives. This engagement helps community members build mutual accountability and connections related to improving health.

### Institutional

At the institutional level, learning collaboratives should partner with local organizations to enhance or expand existing projects. This collaboration can provide additional resources and support, ensuring the sustainability of learning collaborative initiatives, which will aid in ensuring trust within the community. Partnering with local organizations that align with the goals and objectives of the learning collaborative can leverage existing initiatives to increase impact.

### Community

Communities can hold considerable power for change, and learning collaboratives can serve as one avenue through which such change may occur. Within communities, learning collaboratives should ensure that decision-making power is granted to members so that initiatives have local buy-in and are relevant to immediate needs. By focusing on shared goals, objectives, and trust, learning collaboratives can unite diverse community membership in working toward improving overall health. This work could include, for example, partnering with local entities to advocate for food pantries, grocery stores, and sidewalks.

### Policy

Learning collaboratives should be recognized as a public health strategy and used as a tool to guide and influence public health policies. This strategy can begin at the local level through partnerships with local government to advance community initiatives and can extend to integrating learning collaboratives at the national level. Policies should encourage the formation and sustainability of learning collaboratives and provide funding, resources, and training opportunities in both urban and rural communities. With policy support for learning collaboratives, the power for change can be harnessed within communities, leading to improved health outcomes at the individual, community, regional, and national level.

## Conclusion

Learning collaboratives are a powerful public health strategy. Through guiding principles rooted in community organizing, trust, social capital, and shared governance, learning collaboratives can bridge gaps among community members, community partners, public health institutions, and health care organizations. Learning collaboratives can meaningfully address the social, financial, and health care needs of a community through data-driven strategies and by promoting individual empowerment, interpersonal relationships, institutional partnerships, and community engagement. Learning collaboratives and community members can collectively advocate for expanding their impact locally, regionally, and nationally. The strength and success of learning collaboratives lie in their ability to keep community members at the heart of their initiatives, fostering a sense of ownership and collective responsibility in enacting long-lasting change for a healthier tomorrow.
